# Reactive oxygen species generated by irradiation with bandpass-filtered 222-nm Far-UVC play an important role in the germicidal mechanism to *Escherichia coli*

**DOI:** 10.1128/aem.01886-24

**Published:** 2025-01-31

**Authors:** Kouji Narita, Risako Fukushi, Kyosuke Yamane, Yoshihiko Okumura, Toru Koi, Krisana Asano, Akio Nakane

**Affiliations:** 1 Department of Microbiology and Immunology, Hirosaki University Graduate School of Medicine 651535 , Hirosaki, Japan; 2 Institution for Animal Experimentation, Hirosaki University Graduate School of Medicine 12800 , Hirosaki, Japan; 3 Department of Nursing, School of Health Science, Hirosaki University of Health and Welfare 157586 , Hirosaki, Japan; 4 Ushio Inc., Chiyoda-Ku, Tokyo, Japan; 5 Department of Biopolymer and Health Science, Hirosaki University Graduate School of Medicine 12800 , Hirosaki, Japan; Centers for Disease Control and Prevention, Atlanta, Georgia, USA

**Keywords:** filtered 222-nm Far-UVC, reactive oxygen species, cyclobutene pyrimidine dimers, *Escherichia coli*

## Abstract

Ultraviolet (UV) C light emitted by a krypton chloride (KrCl) lamp consists of mainly less harmful 222-nm Far-UVC (unfiltered 222-mm Far-UVC) compared with conventionally used 254-nm UVC. It also contains wavelengths that are harmful to mammalian cells. By contrast, UVC from a KrCl lamp with optical filter (filtered 222-nm Far-UVC) consists of much less harmful 222-nm Far-UVC and is available for sterilization of dwelling spaces. The germicidal mechanisms of the 254-nm UVC and unfiltered 222-nm Far-UVC have been partially elucidated; however, the mechanism of action of filtered 222-nm Far-UVC remains unknown. It is known that 254 nm UVC induces cyclobutene pyrimidine dimers (CPDs), which are DNA lesions in *Escherichia coli* (*E. coli*); however, the CPDs are repaired by photoreactivation. In the present study, it was demonstrated that filtered 222-nm Far-UVC also generated CPDs, which were not repaired by photoreactivation. Therefore, a germicidal mechanism of filtered 222-nm Far-UVC may be different from a 254-nm UVC. It was reported that unfiltered 222-nm Far-UVC induced reactive oxygen species (ROS) in *E. coli*. In the present study, filtered 222-nm Far-UVC also induced ROS production. In accordance with increased ROS production, the levels of carbonylated proteins were increased, and morphological alteration was observed in *E. coli*. From these results, it was suggested that ROS generated by filtered 222-nm Far-UVC inactivated ROS scavenger enzymes and the enzyme photolyase that is involved in photoreactivation. The increased ROS levels and unrepaired CPDs impaired photoreactivation in *E. coli* and may be involved in the germicidal mechanism of action of the filtered 222-nm Far-UVC.

**IMPORTANCE:**

The 222 nm Far-ultraviolet (UV) C light (UVC) emitted from a krypton chloride lamp with an optical filter is currently available for the sterilization of dwelling spaces. To use the filtered 222-nm Far-UVC more effectively and safely for sterilization, it is necessary to understand its germicidal mechanism. The present study suggests that the germicidal effect of filtered 222-nm Far-UVC on *E. coli* may not only involve CPD but also ROS. These results could be useful in establishing more effective preventive methods in dwelling spaces for infectious diseases by UVC irradiation.

## INTRODUCTION

Following the coronavirus disease 2019 pandemic, ultraviolet light (UV) C irradiation has been identified as a new method for disinfection of pathogens present in air and on the surface of materials in public spaces (1, 2). Two hundred fifty-four-nanometer UVC, which is emitted from a low-pressure mercury lamp, elicits a highly germicidal effect and has been conventionally used for disinfection (3). However, it has been documented that a 254-nm UVC is a human health hazard causing dermatitis, keratitis, and increasing risk of skin cancer (4). Recently, 222-nm Far-UVC emitted by a krypton chloride (KrCl) excimer lamp with optical filter (filtered 222-nm Far-UVC) has attracted attention due to the germicidal effect elicited by filtered 222-nm Far-UVC. This effect has been noted for a variety of pathogens, including bacteria, fungi, and viruses, and is similar to that caused by 254-nm UVC (5), yet less harmful to mammalian cells due to absorption by proteins and reduced permeability to biomolecules. Following exposure to the human body, filtered 222-nm Far-UVC is highly absorbed by the stratum corneum of the skin and superficial layer of the corneal limbal epithelium and hardly reaches the basal layer of the dermis and corneal epithelial stem cells (6, 7).

It is known that 254-nm UVC induces formation of mutagenic DNA lesions, with the frequent lesions being cyclobutene pyrimidine dimers (CPDs) and pyrimidine–pyrimidone (6–4) photoproducts (6–4 PPs) that are formed between adjacent pyrimidine bases. These DNA lesions inhibit microbial DNA transcription, translation, and replication and cause bacterial cell death and viral inactivation (8), suggesting that 254-nm UVC elicits its germicidal effect via DNA damage. However, it is reported that bacteria exert the ability to monomerize and repair CPDs and 6–4 PPs by photolyase, which is activated by blue and near-UV light ranging from 350 to 450 nm. This photobiological phenomenon is known as photoreactivation. A part of bacteria inactivated by 254-nm UVC irradiation can recover their ability to proliferate through photoreactivation (9, 10).

Repair efficiency of pyrimidine dimers by photoreactivation depends on the wavelength of UVC used to induce formation of these dimers. It was reported that photoreactivation in bacteria irradiated with 240–580 nm of light emitted from medium-pressure UV lamps was repressed and that the survival rate of the bacteria remains low (11). Similarly, *E. coli* inactivated by 222-nm Far-UVC emitted by the KrCl excimer lamp without optical filter (unfiltered 222-nm Far-UVC) indicated negligible photoreactivation, suggesting that other mechanism(s) should be involved in the inhibition of photoreactivation (12).

Reactive oxygen species (ROS) generated by irradiation with 254-nm UVC has been reported to be involved in the germicidal mechanism by generating DNA damage in *E. coli* (13). When *E. coli* was treated with 254-nm UVC and unfiltered 222-nm Far-UVC simultaneously, the latter inactivated the ROS defense enzyme including superoxide dismutase (SOD) and catalase (CAT). Kang et al. demonstrated that simultaneous irradiation with 222-nm Far-UVC and 254-nm UVC inactivated ROS defense enzymes, resulting in an increase in ROS levels in *E. coli* cells. The increased ROS levels induced greater cell membrane permeability via membrane lipid peroxidation (14). Previous studies on the germicidal mechanism of 222-nm Far-UVC have been reported in unfiltered 222-nm UVC but not in filtered 222-nm Far-UVC.

Unfiltered 222-nm Far-UVC emitted from an KrCl excimer lamp consists of mainly 222-nm Far-UVC light but also contains small amounts of UV light with 230 to 320 nm that are harmful to mammalian skin, whereas filtered 222-nm Far-UVC is less harmful due to harmful wavelength reduction below 0.01% by bandpass filter compared with unfiltered 222-nm Far-UVC (15). Filtered 222-nm Far-UVC is approved for use in disinfection of dwelling spaces, and its use is becoming widespread. However, a germicidal mechanism of filtered 222-nm Far-UVC is not elucidated. In the present study, a germicidal mechanism of filtered 222-nm Far-UVC in *E. coli* was investigated. It was demonstrated that ROS could play an important role in the germicidal mechanism of irradiation with filtered 222-nm Far-UVC.

## RESULTS

### Photoreactivation and dark repair of *E. coli* irradiated with 254-nm UVC or filtered 222-nm Far-UVC

**Fig 1:**
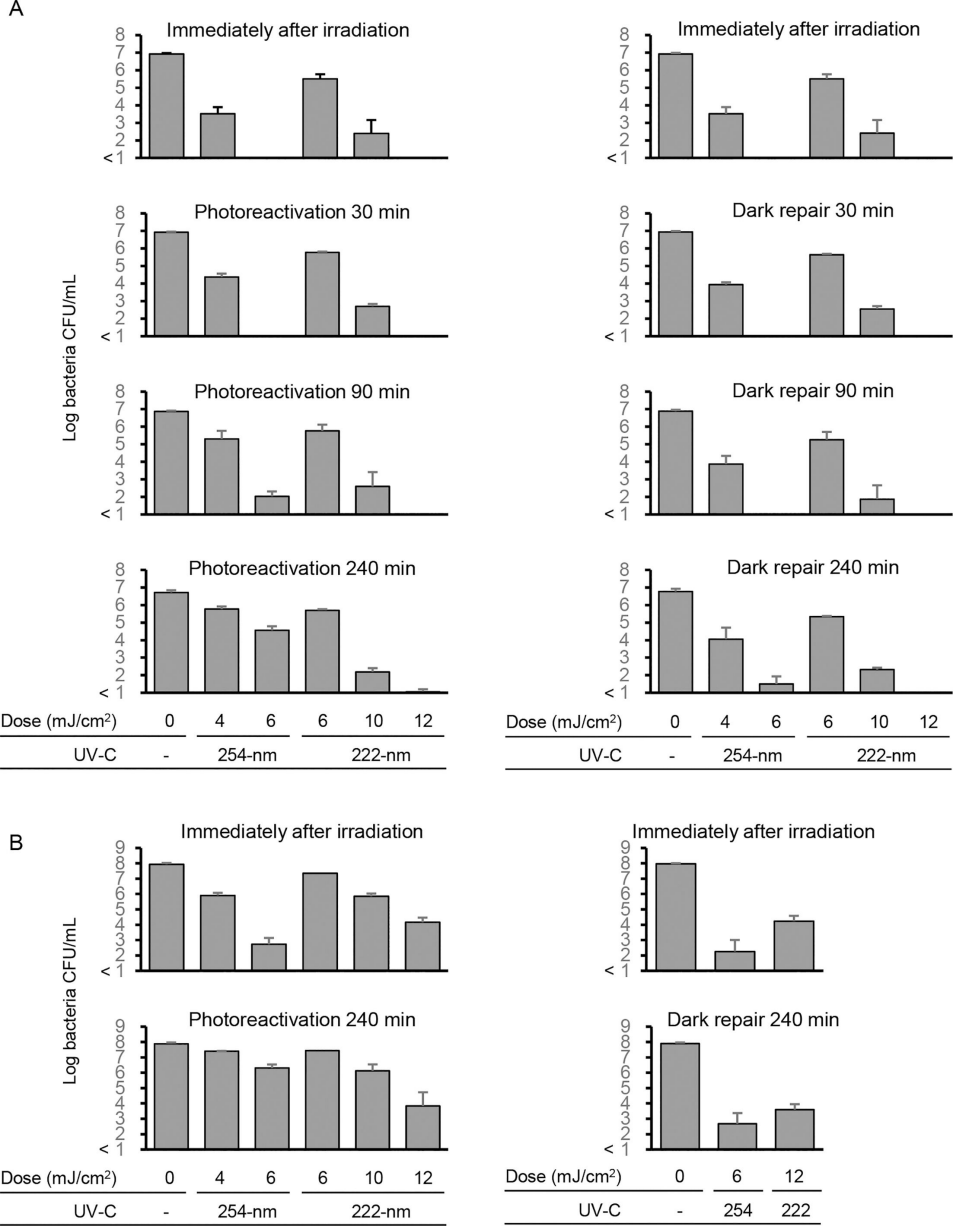
Bacterial count of *E. coli* following irradiation with 254-nm or filtered 222-nm Far-UVC and following photoreactivation or dark repair. *E. coli* suspension at 1 × 10^7^ CFU/mL (**A**) and 1 × 10^8^ CFU/mL (**B**) was irradiated with 254-nm or filtered 222-nm Far-UVC and then left to stand under light and dark condition for indicated minutes to induce photoreactivation and dark repair, respectively. Data are expressed as means ± standard deviation.

Following irradiation of the *E. coli* suspension [1 × 10^7^ colony-forming units (CFUs)/mL] with 254-nm UVC at 4 and 6 mJ/cm^2^, the number of viable bacteria was reduced from log 6.9 to log 3.5 and <log 1, respectively. Following photoreactivation for 240 min, the count of the cultured bacteria was increased to log 5.8 and log 4.6, respectively (Fig. 1A). Irradiation of the bacterial suspension (1 × 10^8^ CFU/mL) with 254-nm UVC at 4 and 6 mJ/cm^2^ resulted in the recovery of viable *E. coli* (Fig. 1B). Irradiation with filtered 222-nm Far-UVC at 6, 10, and 12 mJ/cm^2^ to bacterial suspensions at a concentration of 1 × 10^7^ CFU/mL reduced cultured *E. coli* from log 6.9 to log 5.4, log 2.2 and <log 1, respectively (Fig. 1A). Irradiation with filtered 222-nm Far-UVC at 10 and 12 mJ/cm^2^ to bacterial suspensions (concentration of 1 × 10^8^ CFU/m) also inactivated *E. coli* from log 7.9 to log 5.9 and log 4.2, respectively (Fig. 1B). In contrast to the irradiation with 254-nm UVC, the count of bacterial cells irradiated with filtered 222-nm Far-UVC was comparable between that noted immediately following irradiation and that noted following photoreactivation treatment for 240 min, indicating that *E. coli* irradiated with filtered 222-nm Far-UVC could not recover by the photoreactivation treatment (Fig. 1A and B). When *E. coli* was irradiated with either 254-nm or filtered 222-nm Far-UVC, the bacterial count was comparable with that noted prior to and following dark repair treatment (Fig. 1A and B), indicating that neither *E. coli* irradiated with 254-nm UVC nor *E. coli* irradiated with 222-nm Far-UVC could recover via dark repair.

### CPD formation in *E. coli* following irradiation with 254-nm or filtered 222-nm Far-UVC and following photoreactivation or dark repair

**Fig 2:**
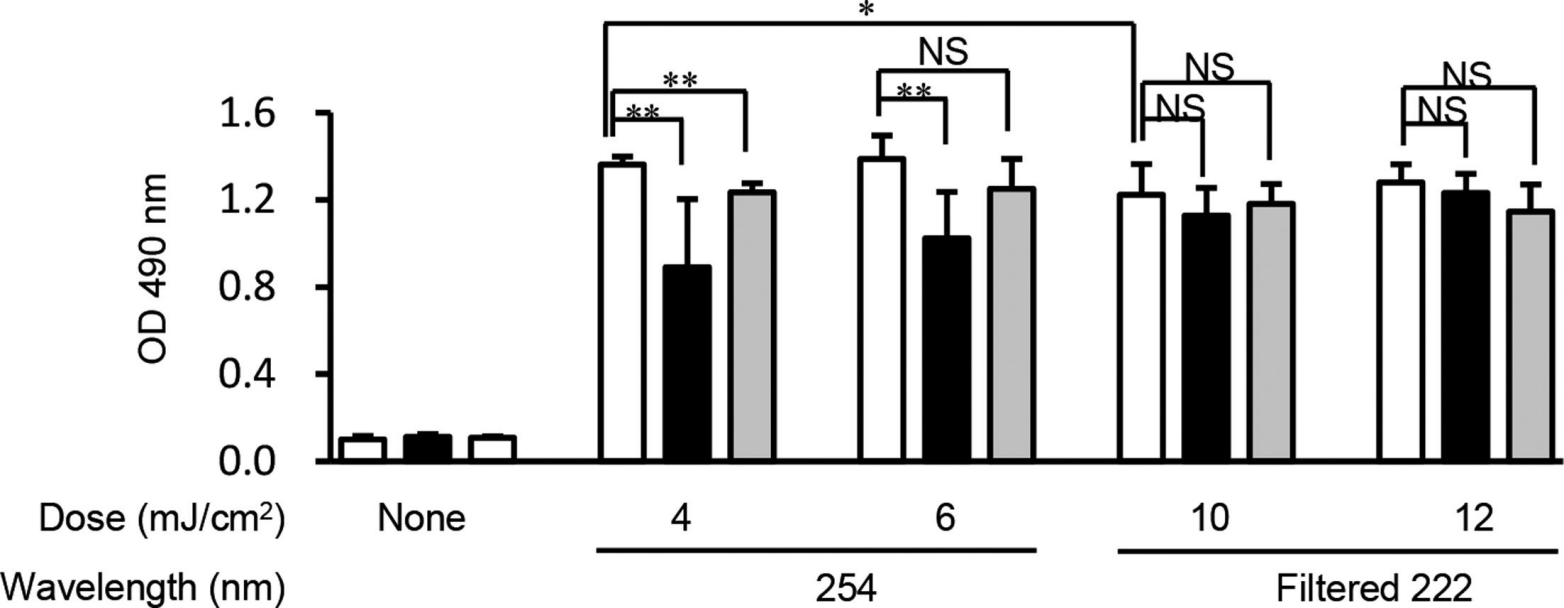
CPD formation in *E. coli* following irradiation with 254-nm or filtered 222-nm Far-UVC and following photoreactivation or dark repair. *E. coli* suspension at 1 × 10^7^ CFU/mL was non-irradiated, irradiated with 254-nm UVC with 4 or 6 mJ/cm^2^, and filtered 222-nm Far-UVC with 10 or 12 mJ/cm^2^. CPD was quantified immediately following irradiation (open bar), following photoreactivation (black bar), or dark repair for 240 min (gray bar) by ELISA. Data are expressed as the means ± standard deviation. NS indicates not significant. Single and double asterisks indicate statistical significance at *P* < 0.05 and *P* < 0.01, respectively.

Irradiation with 254-nm or filtered 222-nm Far-UVC induced a significant amount of CPDs in *E. coli*. This effect was noted immediately following irradiation compared with the non-irradiated *E. coli* culture (Fig. 2). Irradiation with 254-nm UVC at 4 mJ/cm^2^ and with filtered 222-nm Far-UVC at 10 mJ/cm^2^ significantly induced CPD formation in *E. coli*, although the CPD levels of *E. coli* irradiated with 254-nm UVC at 4 mJ/cm^2^ were significantly higher than those noted with filtered 222-nm Far-UVC at 10 mJ/cm^2^ (Fig. 2). CPD formation was induced by irradiation with 254-nm UVC at 4 and 6 mJ/cm^2^ and was significantly reduced following photoreactivation for 240 min. In contrast to these observations, CPD formation in *E. coli* irradiated with filtered 222-nm Far-UVC at 10 and 12 mJ/cm^2^ was comparable prior to and following photoreactivation treatment for 240 min. (Fig. 2).

### ROS generation in *E. coli* following irradiation with 254-nm or with filtered 222-nm Far-UVC and following photoreactivation or dark repair

**Fig 3:**
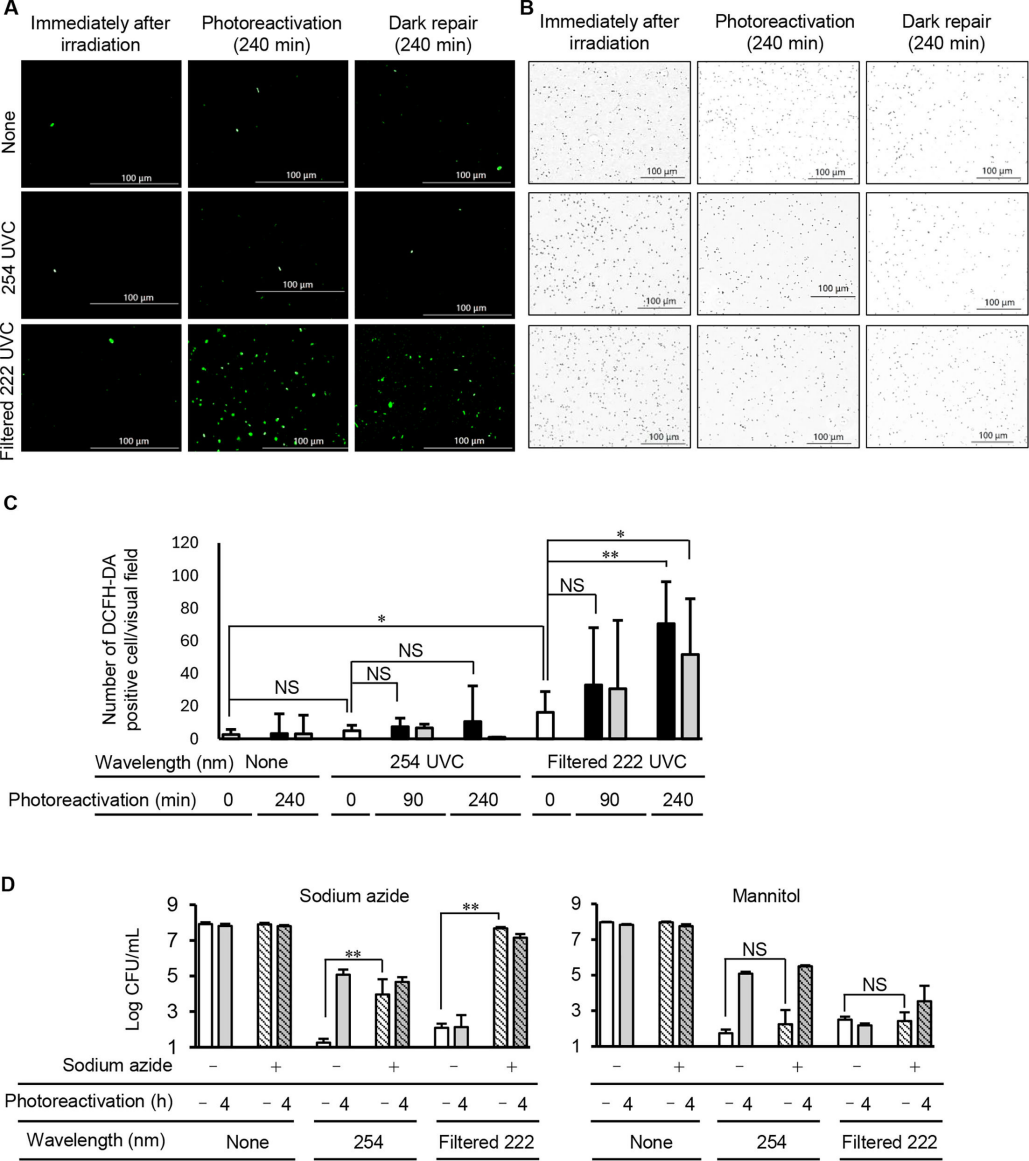
ROS generation in *E. coli* following irradiation with 254-nm or filtered 222-nm Far-UVC and following photoreactivation or dark repair. *E. coli* was irradiated with 254-nm UVC at 6 mJ/cm^2^ or filtered 222-nm Far-UVC at 12 mJ/cm^2^, or non-irradiated and then photoreactivated or dark repaired for 0, 90, or 240 min. (**A**) ROS-generating *E. coil* were stained by DCFH-DA; (**B**) the bright-field images. (**C**) Count of ROS-generating *E. coli* cell irradiated with or without UVC and then non-reactivated (open bar), photoreactivated (black bar), or dark repaired (gray bar) for 90 or 240 min. The average number of DCFH-DA-positive cells was obtained by calculating the average value of at least three visual fields (×400). Scale bar = 100 µm. Data are expressed as the means ± standard deviation. (**D**) Bacterial count of *E. coli* pretreated with sodium azide or mannitol (hatched bar) immediately following filtered 222-nm Far-UVC (open bar) and following photoreactivation for 240 min (4 h) (gray bar). NS indicates not significant. Single and double asterisks indicate statistical significance at *P* < 0.05 and *P* < 0.01, respectively.

The following *E. coli* cultures were used: *E. coli* irradiated with 254-nm UVC at 6 mJ/cm^2^, with filtered 222-nm Far-UVC at 12 mJ/cm^2^, or non-irradiated. These groups were subsequently photoreactivated or dark repaired for 0, 90, or 240 min. ROS levels in *E. coli* were detected by 2′,7′-dichlorodihydrofluorescein diacetate (DCFH-DA), which is a green fluorescent dye used as a probe for the detection of intracellular ROS. The 254-nm UVC did not induce ROS generation in *E. coli* immediately following irradiation. and ROS-producing cells were comparable prior to and following photoreactivation or dark repair treatment for 90 and 240 min (Fig. 3A and C). By contrast, irradiation with filtered 222-nm Far-UVC induced ROS formation in *E. coli* cells immediately following irradiation, and the ROS content was gradually increased following photoreactivation and dark repair treatment for 90 and 240 min (Fig. 3A and C). In bright-field images, the cell counts of non-irradiated *E. coli* and *E. coli* irradiated with UVC were comparable immediately following irradiation; this count was also similar following photoreactivation and dark repair (Fig. 3B)

### Inactivation of *E. coli* treated with different ROS scavengers by 254-nm UVC or filtered 222-nm Far-UVC

Sodium azide and mannitol indicated no influence to the viability of *E. coli*. Sodium azide partially reduced the treatment lethality by irradiation with 254-nm UVC on *E. coli*, and photoreactivation was not observed, whereas drug treatment inhibited the germicidal effect by irradiation with filtered 222-nm Far-UVC. By contrast, no significant effect was observed in either 254-nm or filtered 222-nm Far-UVC when mannitol was used (Fig. 3D).

### Protein carbonylation in *E. coli* following irradiation with 254-nm UVC or filtered 222-nm Far-UVC and following photoreactivation or dark repair

**Fig 4:**
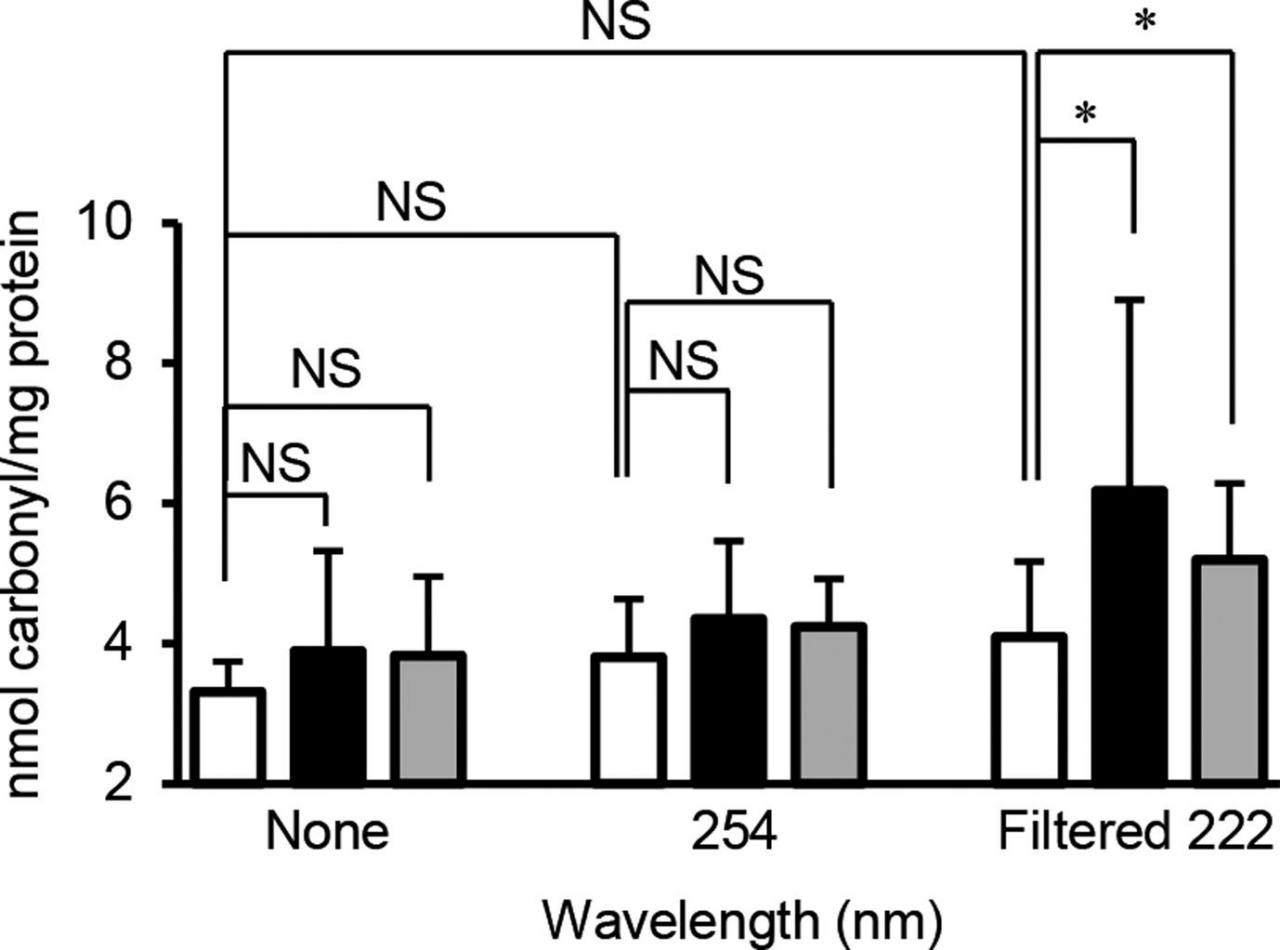
Protein carbonylation in *E. coli* following irradiation with 254-nm or filtered 222-nm Far-UVC and following photoreactivation or dark repair. *E. coli* was non-irradiated or irradiated with 254-nm UVC at 6 mJ/cm^2^ or filtered 222-nm Far-UVC at 12 mJ/cm^2^ and then non-reactivated (open bar), photoreactivated (black bar), or dark repaired (gray bar) for 240 min. Protein carbonylation was measured as described in Materials and Methods. Data are expressed as the means ± standard deviation. NS indicates not significant. An asterisk indicates statistical significance at *P* < 0.05.

ROS attack proteins and cause irreversible carbonylation that is a marker of protein oxidation. It was reported that the levels of protein carbonylation in *E. coli* exposed to UVC light correlated with cell killing (16). Therefore, protein carbonylation was measured in *E. coli* irradiated with 254-nm or filtered 222-nm Far-UVC immediately following irradiation and following photoreactivation or dark repair for 240 min. Neither 254-nm nor filtered 222-nm Far-UVC caused protein carbonylation immediately following the irradiation, whereas protein carbonylation was increased in *E. coli* irradiated with filtered 222-nm Far-UVC following photoreactivation and dark repair for 240 min (Fig. 4). The increase in protein carbonylation was detected in neither non-irradiated nor 254-nm UVC irradiated *E. coli* following photoreactivation and dark repair (Fig. 4).

### Scanning electron microscopy (SEM) images of 254-nm or filtered 222-nm Far-UVC-irradiated *E. coli* following photoreactivation

**Fig 5:**
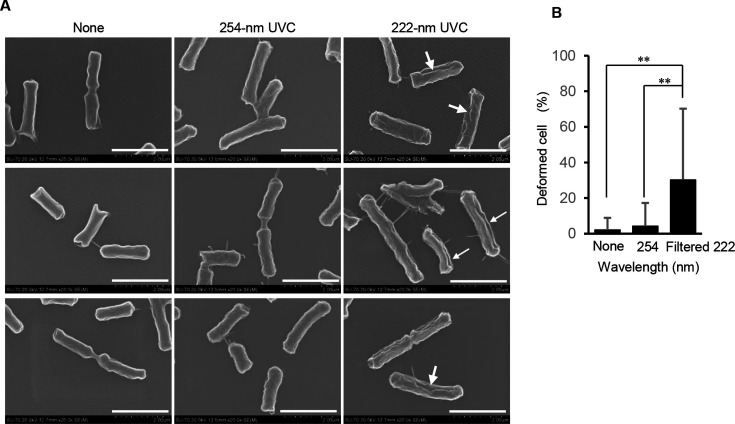
SEM images of *E. coli* irradiated with 254-nm or filtered 222-nm Far-UVC following photoreactivation. SEM observation (×20,000) of morphology changes in *E. coli* cells non-irradiated or irradiated with 254-nm UVC at 6 mJ/cm^2^ or filtered 222-nm Far-UVC at 12 mJ/cm^2^ and then photoreactivated for 4 h (**A**). Arrows indicate distorted bacterium. Scale bar = 2 µm. (**B**) Percentage of distorted bacteria. Double asterisk indicates statistical significance at *P* < 0.01.

Unfiltered 222 nm Far-UVC irradiation was reported to cause cell membrane damage and deformation in the cell shape of *E. coli* (12). To investigate the possibility of filtered 222-nm Far-UVC to deform the cell shape of *E. coli*, the morphology of *E. coli* treated with 254-nm or filtered 222-nm Far-UVC was observed using SEM. Non-irradiated *E. coli* cells exhibited a rod-like shape with a slightly wrinkled surface. The morphology of *E. coli* cells irradiated with 254-nm UVC at 6 mJ/cm^2^ was similar to that of non-irradiated *E. coli* cells; however, they appeared to exhibit slightly increased surface wrinkles. In contrast to these observations, the morphology of *E. coli* cells irradiated with filtered 222-nm Far-UVC at 12 mJ/cm^2^ was clearly wrinkled and uneven, with large dents on the cell surface (Fig. 5).

## DISCUSSION

The present study indicated that the germicidal effect of filtered 222-nm Far-UVC was lower than that of 254-nm UVC (Fig. 1A). By contrast, previous reports documented that unfiltered 222-nm Far-UVC elicited higher germicidal effect on certain bacterial types compared to the effect noted with 254-nm UVC (14, 17). The reason for this discrepancy between filtered 222-nm and unfiltered 222-nm Far-UVC may be due to the different spectra. Unfiltered 222-nm Far-UVC contains a small level of 230- to 320-nm UVC, and these wavelengths may be involved in the germicidal effect. By contrast, these wavelengths are reduced approximately below 0.01% in the spectra of filtered 222-nm Far-UVC (15).

*E. coli* cells inactivated by 254-nm UVC could recover their proliferative activity by photoreactivation (9, 10). However, bacteria inactivated by unfiltered 222-nm Far-UVC could not recover by photoreactivation treatment (12). Similar to unfiltered 222-nm Far-UVC, *E. coli* irradiated with filtered 222-nm Far-UVC did not recover its proliferative ability by photoreactivation (Fig. 1).

The amount of CPD in 254-nm UVC-irradiated *E. coli* cells was slightly higher than that noted in filtered 222-nm Far-UVC-irradiated cells. This tendency coincided with the previous reports using unfiltered 222-nm Far-UVC (12, 17). CPDs in *E. coli* irradiated with 254-nm UVC were reduced by photoreactivation, while the amount of CPD in *E. coli* irradiated with filtered 222-nm Far-UVC was not affected by photoreactivation as previously reported using unfiltered 222-nm Far-UVC (12, 18) (Fig. 2). These results support the conclusion that CPD unrepaired by photoreactivation may be involved in the continuous germicidal effect of filtered 222-nm Far-UVC. By contrast, CPD was not repaired following dark repair treatment (Fig. 2), and this result was consistent with the effects noted on the impaired proliferative ability noted in *E. coli* cells (Fig. 1).

It is known that 254-nm UVC generated ROS that caused oxidative damage in culture and *E. coli* cells (13, 19, 20). ROS induce formation of other oxygen radicals that can cause oxidative damage of lipids, proteins, and DNA in pathogens (21). It was reported that unfiltered 222-nm Far-UVC induced intracellular ROS generation in a similar manner as 254-nm UVC (12, 14, 17). In the present study, filtered 222-nm Far-UVC also induced ROS generation in *E. coli* cells immediately following irradiation, and the ROS-generating cells were increased during photoreactivation and dark repair (Fig. 3).

ROS include superoxide, hydroxyl radical, singlet oxygen, and hydrogen peroxide. Singlet oxygen generated by UVB is reported to play an important role in the germicidal effect by inducing lipid peroxidation (22). In the present study, sodium azide, which is a singlet oxygen scavenger, partially reduced the germicidal effect of 254-nm UVC immediately following irradiation and inhibited the proliferative activity of *E. coli* cells by photoreactivation (Fig. 3D). These results indicated that not only CPD but also singlet oxygen was involved in the germicidal effect of 254-nm UVC. By contrast, ROS were not detected in *E. coli* cells irradiated with 254-nm UVC (Fig. 3A and C). Singlet oxygen is one of the most reactive ROS and can induce oxidative damage at a quite low dose. Therefore, singlet oxygen may act below a detectable level of ROS. It was reported that unfiltered 222-nm Far-UVC inactivated *E. coli* and damaged the cell membrane in the presence of D-mannitol, which is a hydroxyl radical scavenger (12). In the present study, D-mannitol exhibited a minimal effect on the germicidal effect of 254-nm and filtered 222-nm Far-UVC (Fig. 3D). When 222-nm Far-UVC irradiation was performed in the presence of sodium azide, the germicidal effect of 222-nm Far UVC was not observed immediately following irradiation. By contrast, the germicidal effect remained evident in the absence of mannitol, suggesting that singlet oxygen plays a role in this process.

ROS attack protein and cause irreversible protein oxidation resulting in protein carbonylation that affects the synthesis and conformational processes of proteins and leads to inactivated enzyme activity (23–25). In the present study, the carbonylated protein in *E. coli* cells irradiated with filtered 222-nm Far-UVC was increased following photoreactivation and dark repair (Fig. 4). The major ROS defense enzymes include SOD and CAT; these enzymes were reported to be inactivated by ROS (26, 27). It is possible that ROS produced in *E. coli* cells by irradiation with filtered 222-nm Far-UVC caused carbonylation and inactivation of ROS scavenging enzymes, whereas ROS are further produced via aerobic metabolism in *E. coli* cells; moreover, increasing ROS generation further inactivates ROS removal enzymes, leading to cell damage.

The abnormal morphology of *E coli* cells was induced by irradiation with filtered 222-nm Far-UVC but not with 254-nm UVC (Fig. 5). ROS interact with bacterial cell membranes and impair their selective permeability by causing lipid peroxidation that is harmful to the cell membrane structure (28, 29). It was reported that ROS increased the cell membrane permeability of *Campylobacter jejuni*, and the helical morphology of the bacterium was altered to acquire a globular shape (29). In addition, ROS were also reported to cause morphological alterations in *E. coli* and *Helicobacter pylori* (12, 30, 31). From these studies, it may be possible to deduce that ROS produced by irradiation with filtered 222-nm Far-UVC as well as unfiltered 222 nm Far-UVC can cause peroxidation of lipids in the cell membrane leading to a morphological alteration in *E. coli* cells.

In the present study, it was demonstrated that filtered 222-nm Far-UVC induced not only pyrimidine dimer formation in DNA but also ROS generation, notably singlet oxygen generation; in addition, ROS induced protein carbonylation, resulting in *E. coli* cell death. It is possible that ROS scavenger enzymes are inactivated by protein carbonylation resulting in an increase in ROS levels, which inhibit repair of CPD by photoreactivation. It has been reported that 222-nm Far-UVC exhibits germicidal effects on aerosols of *E. coli* deposited on various material surfaces, including cotton carpets, acrylic, wood, cotton towels, and glass commonly found in dwelling spaces (32). In the present study, the germicidal mechanisms of filtered 222-nm Far-UVC were investigated using *E. coli* suspended in phosphate-buffered saline (PBS) to investigate in a stable condition. However, it is possible that ROS are involved in the germicidal mechanisms against *E. coli* in living environments. Although further research is required to elucidate the germicidal mechanism of filtered 222-nm Far-UVC, ROS induced by filtered 222-nm Far-UVC irradiation may play an important role in the germicidal mechanism to *E. coli*.

## MATERIALS AND METHODS

### Bacterial strain and culture condition

*E. coli* ATCC 25922 was cultured in tryptic soy broth (BD diagnosis Systems, Sparks, MD, USA) at 37°C for 12–14 h and harvested by centrifugation at 1,100 × *g* for 10 min at 4°C. The bacterial cells were washed with sterile PBS three times and subsequently diluted with PBS to an appropriate concentration by spectrophotometric measurement at 550 nm using a spectrophotometer (UV-1900i, Shimadzu, Kyoto, Japan).

### UVC light and fluorescent light sources

To irradiate UVC light, the two following types of lamp devices were used: The first device was the Care222 (Ushio Inc. Tokyo, Japan); it consists of a KrCl excimer lamp and an optical filter and emits almost a single wavelength of 222-nm Far-UVC. The spectra emitted from the device were measured by a multichannel spectrometer QEP01172 (Ocean Insight, Orlando, FL, USA). The QEP 01172 was calibrated with the L7820 D2 lamp (Hamamatsu Photonics K.K., Hamamatsu, Japan) by the National Institute of Advanced Industrial Science and Technology. The spectra emitted from this device used in the present study are shown in Fig. S1A and S1B. The second device was the SUV-4 (As One Co., Ltd., Osaka, Japan), which consists of conventional low-pressure mercury lamp and emits a line spectrum of 254 nm. An integrated UV meter S-172/UIT250 (Ushio) was used to measure the irradiance of UVC lights using coefficients for each wavelength. The irradiance was set to 0.05 mW/cm^2^ by adjusting the distance from the irradiator window. For photoreactivation, fluorescent lighting fixture, Sky Light Slim (KotobukiKogeiCo., Ltd., Nara, Japan) was used, which was installed with a fluorescent lamp FL20SSENW18HF2 (Panasonic Co., Ltd., Tokyo, Japan). The spectral irradiance of this lamp was measured in the biological safety cabinet by lighting the fluorescent lamp attached to the cabinet (Panasonic). The distance between the fluorescent lighting fixture for photoreactivation and the light-receiving window of spectrometer USR-45DA-14(1509001) (Ushio) was 11 cm. The spectral irradiance is shown in Fig. S1C. The integrated value of the spectral illuminance of 300 to 500 nm was 563.9 to 592.5 μW/cm^2^ within 30 min following lighting.

### Count of bacteria immediately following UVC irradiation and following photoreactivation or dark repair

A total of 20 mL of *E. coli* suspension at a concentration of 1 × 10^7^ or 1 × 10^8^ CFU/mL was used in a Petri dish with a diameter of 90 mm. The culture was non-irradiated or irradiated with either 254-nm UVC at 4 and 6 mJ/cm^2^ or with filtered 222-nm Far-UVC at 6, 10, and 12 mJ/cm^2^, respectively, using the UVC light devices described above.

To induce photoreactivation, bacterial suspension was placed at a distance of 11 cm from a fluorescent lighting fixture in a biological safety cabinet equipped with the fluorescent lamp turned on and illuminated with a fluorescent light for 30, 90, and 240 min. For dark repair, the bacterial suspension was allowed to stand in the dark for the same time period. To enumerate the bacterial count in the bacterial suspension immediately following UVC irradiation and following photoreactivation or dark repair, the bacterial suspension was serially diluted 10-fold, and an aliquot of 0.1 mL was transferred to a tryptic soy agar (BD diagnosis Systems). The colonies were counted at 24 h following incubation at 37°C.

### Detection of CPD in *E. coli*

A total of 20 mL *E. coli* suspension at a concentration of 1 × 10^7^ CFU/mL was transferred to a Petri dish with a diameter of 90 mm. The culture was non-irradiated or irradiated with 254-nm UVC at 4 and 6 mJ/cm^2^ or with 222-nm Far-UVC at 10 and 12 mJ/cm^2^. Immediately following UVC irradiation and following photoreactivation or dark repair for 240 min, genomic DNA was isolated from the *E. coli* culture using NucleoSpin DNA RapidLyse (Takara Bio, Shiga, Japan) according to the manufacturer’s instructions. The concentration of the DNA was measured spectrophotometrically using a NanoDrop Lite (Thermo Scientific, Waltham, MA, USA). ELISA was performed to determine the quantities of CPD in the DNA samples using an anti-CPD monoclonal antibody (Clone TDM-2) (Cosmo Bio, Ltd., Tokyo, Japan) according to the manufacturer’s instructions with some modifications. Briefly, 96-well ELISA plates were coated with 0.003% protamine sulfate, and 50 µL of heat-denatured DNA samples (1 µg/mL) was distributed. Following blocking with 2% fetal bovine serum in PBS, an anti-CPD monoclonal antibody was added and incubated for 30 min at 37°C; subsequently, a horseradish peroxidase-conjugated goat anti-mouse IgG2a antibody (Southern Biotechnology Associates, Inc., Birmingham, AL, USA) was added and incubated for 2 h at 37°C. The absorbance of a colored product derived from *o*-phenylene diamine was measured at 490 nm using a spectrophotometer (UV-1900i, Shimadzu, Kyoto, Japan).

### Detection of ROS in *E. coli*

ROS levels in *E. coli* were detected using a ROS Assay Kit—Highly Sensitive DCFH-DA (Dojindo Co., Ltd., Kumamoto, Japan) according to the manufacturer’s instructions with some modifications. Briefly, 1 mL of *E. coli* suspension at a concentration of 1 × 10^8^ CFU was centrifuged at 8,000 × *g* for 10 min at 4°C and resuspended in 100 µL working solution containing DCFH-DA. The resuspension was non-irradiated or irradiated with 254-nm UVC at 6 mJ/cm^2^ or with filtered 222-nm Far-UVC at 12 mJ/cm^2^. Immediately following UVC irradiation and following photoreactivation or dark repair for 90 and 240 min, an aliquot of 15 µL was placed on a glass slide. The ROS levels in the *E. coli* cells were observed using a BZ-X700 fluorescence microscope (Keyence, Osaka, Japan), and the DCFH-DA positive cells were counted automatically using a BZ-X800 Analyzer (Keyence).

### Detection of protein carbonylation in *E. coli*

A total of 20 mL *E. coli* suspension (concentration of 1 × 10^8^ CFU/mL) was cultured in a Petri dish with a diameter of 90 mm. The suspension was non-irradiated or irradiated with 254-nm UVC at 6 mJ/cm^2^ or with filtered 222-nm Far-UVC at 12 mJ/cm^2^. Immediately following UVC irradiation and following photoreactivation or dark repair for 240 min, the bacterial suspension was centrifuged at 8,000 × *g* for 10 min at 4°C, and the supernatant was removed. The protein was extracted from the bacteria using 200 µL B-PER bacterial protein extraction reagent (Thermo Scientific), and 100 µL 2,4-dinitrophenylhydrazine (DNPH, Fujifilm Wako Pure Chemical Co.) solution (10 µM) was added to each sample and incubated for 1 h. A total of 60 µL trichloroacetic acid solution (100%) was added and incubated on ice for 5 min, and the supernatant was removed following centrifugation at 13,000 × *g* for 5 min at 4°C. The pellet was washed with 1 mL ice-cold acetone to remove free DNPH. A total of 200 mL 6 M guanidine hydrochloride (Fujifilm Wako) solution was added, and the pellets were resolubilized. A total of 100 µL of each sample was transferred to a 96-well microplate, and the concentration of the protein carbonylation was measured spectrophotometrically at 375 nm using a molar absorption coefficient of 22,000 M^−1^ cm^−1^ (33).

### Treatment with ROS scavengers

Sodium azide (Fujifilm Wako) and D(−)-mannitol (Fujifilm Wako) are specific ROS scavengers of singlet oxygen and hydroxyl radical, respectively (22). These antioxidants were dissolved in PBS at a concentration of 10 mM. *E. coli* cells were pre-incubated with the antioxidants for 30 min with agitation. Consequently, *E. coli* cells were washed and resuspended in PBS at a concentration of 1 × 10^8^ CFU/mL. A total of 20 mL of prepared suspensions were irradiated with 254-nm UVC or filtered 222-nm Far-UVC at 6 and 12 mJ/cm^2^, respectively.

### SEM observation

A total of 3 mL of *E. coli* suspension at a concentration of 1 × 10^8^ CFU/mL was placed in a 35-mm dish coated with 0.01% poly-L-lysine and incubated for 90 min to allow the bacteria to attach to the dish. The *E. coli* suspension was non-irradiated or irradiated with either 254-nm UVC at 6 mJ/cm^2^ or filtered 222-nm Far-UVC at 12 mJ/cm^2^. Immediately following UVC irradiation and following photoreactivation or dark repair for 240 min, the bacterial suspension was removed, and 3 mL of glutaraldehyde (2.5%) was added to the dish to fix the bacteria for 90 min. Following fixation, the dish was washed three times with distilled water, gradually dehydrated with ethanol (50%, 70%, 80%, 90%, 95%, and 100%) for 10 min each and dried. The morphology of *E. coli* cells was observed with SEM. Briefly, the samples were processed using a carbon evaporator (approximately 50 nm carbon) VC-100 (Vacuum Device Inc., Ibaraki, Japan). The bacterial samples were observed using a scanning electron microscope SU-70 (Hitachi High-Tech Co., Tokyo, Japan). The percentage of deformed bacteria was quantified by counting the deformed cells in 20 SEM images.

### Statistical analysis

All experiments were performed in triplicate. The data are expressed as mean ± standard deviation. Statistical analyses were performed using Student’s *t*-test. *P* < 0.05 was considered to indicate a statistically significant difference.

## SUPPLEMENTAL MATERIAL

Figure S1 aem.01886-24-s0001.tif Spectral irradiance of filtered and unfiltered KrCl excimer lamp and lamp of a fluorescent lighting fixture.

ASM does not own the copyrights to Supplemental Material that may be linked to, or accessed through, an article. The authors have granted ASM a non-exclusive, world-wide license to publish the Supplemental Material files. Please contact the corresponding author directly for reuse.
